# Epithelial–stromal cell interactions and extracellular matrix mechanics drive the formation of airway-mimetic tubular morphology in lung organoids

**DOI:** 10.1016/j.isci.2021.103061

**Published:** 2021-08-30

**Authors:** Tankut.G. Güney, Alfonso Muinelo Herranz, Sharon Mumby, Iain E. Dunlop, Ian M. Adcock

**Affiliations:** 1 Airways Disease Section, National Heart and Lung Institute, Imperial College London, Dovehouse Street, London SW3 6LY, UK; 2 Department of Materials, Imperial College London, Exhibition Road, London, UK; 3 Helmholtz Zentrum Muenchen, Deutsches Forschungszentrum für Gesundheit und Umwelt (GmbH), Ingolstaedter Landstraße 1D, Neuherberg, München 85764, Germany

**Keywords:** Bioengineering, Tissue Engineering, Biotechnology, Biomechanics

## Abstract

Complex human airway cellular organization where extracellular matrix (ECM) and epithelial and stromal lineages interact present challenges for organ study *in vitro*. Current *in vitro* lung models that focus on the lung epithelium do not represent complex airway morphology and cell-ECM interactions seen *in vivo*. Models including stromal populations often separate them via a semipermeable barrier precluding cell–cell interaction or the effect of ECM mechanics. We investigated the effect of stromal cells on basal epithelial cell-derived bronchosphere structure and function through a triple culture of human bronchial epithelial, lung fibroblast, and airway smooth muscle cells. Epithelial–stromal cross-talk resulted in epithelial cell-driven branching tubules with stromal cells surrounding epithelial cells termed bronchotubules. Agarose– Matrigel scaffold (Agrigel) formed a mechanically tuneable ECM, with adjustable viscoelasticity and stiffness enabling long-term tubule survival. Bronchotubule models may enable research into how epithelial–stromal cell and cell–ECM communication drive tissue patterning, repair, and development of disease.

## Highlights

• Healthy lung epithelial and fibroblast cell coculture in Matrigel forms tubules
• Tubules collapse in 4 days
• Addition of healthy airway smooth muscle cells allows for a contractile phenotype
• Triple culture in stiffer matrix maintains tubular organoid structure for 20 days

## Graphical abstract

**Figure:**
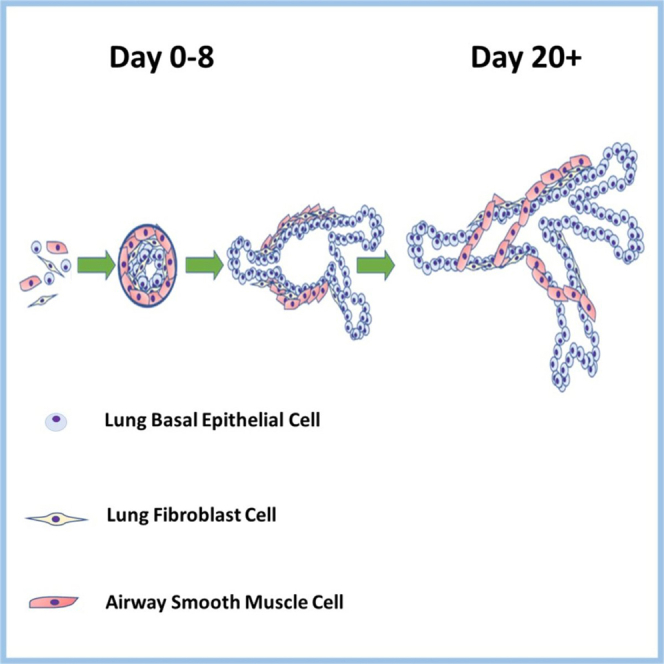

## Introduction

The airway is a complex organ consisting of highly branched tubes that provide air to the alveoli enabling gas exchange. These tubes contain multiple layers of interacting cells and the development, maintenance, and regeneration of the airway is governed by the cell–cell and cell–extracellular matrix (ECM) interactions. Furthermore, the mechanical properties of the ECM in the lower airways are integral to tissue architecture and cellular differentiation (Zhou et al., 2018; Burgstaller et al., 2017; Bonnans et al., 2014).

The airway is formed from a pseudostratified epithelial layer of mucous and ciliated cells underlined by basal cells attached to a basement membrane ECM (Hogan et al., 2014; Rock et al., 2010). The basement membrane separates the epithelium from the interstitial matrix (lamina propria) that contains immune and fibroblast cells as well as vasculature (Hogan et al., 2014; Rock et al., 2010). Fibroblasts provide structural support by secreting ECM as well as being a main player during cell and ECM repair (White, 2015). Bands of airway smooth muscle form around the lamina propria and control the size of the airway lumen (Doeing and Solway, 2013). Perturbation of cell-cell and cell-ECM mechanical dynamics is associated with diseases such as interstitial pulmonary fibrosis (IPF) or chronic obstructive pulmonary disease (COPD) (Zhou et al., 2018; Burgstaller et al., 2017; Bonnans et al., 2014; Volckaert et al., 2013; Yuan et al., 2018).

Monolayer cultures of airway epithelium are ineffective in modeling disease reflecting the differences by which primary human cells interact with each other and how they communicate within the airway 3-dimensional (3D) structure (Gkatzis et al., 2018; Barkauskas et al., 2013). As a result, more complex models including lung-on-a-chip have been developed (Gkatzis et al., 2018; Benam et al., 2016; Osei et al., 2016), that provide an opportunity to expose cells to environmental stimuli and/or examine the impact of immune cells (Benam et al., 2016). However, these 2-dimensional models are not representative of the *in vivo* airway environment where neighboring cells physically interact with each other to form tissue within a 3D space (Benam et al., 2016; Huh et al., 2010).

To address this gap 3D organoids have been developed using stem or stem-like cells that self-organize and autodifferentiate in the presence of an ECM scaffold to form the 3D functional multilineage architecture of the originating organ tissue (Sato et al., 2011; Barker et al., 2010; Danahay et al., 2015; Hindley et al., 2016). Lung organoids or organospheres form from the aggregation and differentiation of human bronchial or tracheal basal cells that give rise to multiple cell lineages including ciliated and goblet cells (Danahay et al., 2015; Broutier et al., 2017; Barkauskas et al., 2017). Lung organoids were originally derived from mouse tracheal basal cells, whilst later studies used human bronchial basal epithelial cells to demonstrate that Notch2 and IL-13 inhibition enhanced the proportion of *Foxj1*-positive ciliated cells at the expense of mucous producing cells (Danahay et al., 2015).

The use of human pluripotent stem cells has enabled the recapitulation of the tubular architecture of the airways. These lung bronchial organoids form in ∼170 days and recapitulate the features of the embryonic lung showing alveolar ATI and ATII markers distally and the club and mucous cell markers SCGB3A2 and MUC5AC proximally (Chen et al., 2017). However, these models do not contain mesenchymal populations such as fibroblasts or airway smooth muscle (ASM) cells that are crucial for the development and maintenance of the airway, for airway contraction and remodeling and are dysregulated in airway diseases such as asthma, COPD, and IPF (Bitterman, 2018; Miglino et al., 2012; Michalik et al., 2018). In addition, the effects of the ECM released by mesenchymal cells need to be considered as a key parameter in organoid design, since ECM mechanics profoundly affect cellular behavior, differentiation, and overall organ morphology (Marinkovic et al., 2013; Piccolo et al., 2014; El Agha et al., 2017).

Biomaterials and tissues exhibit complex mechanical properties that are not always well-summarized by a single number such as Young’s Modulus. Biomaterials are often viscoelastic, with both viscous and elastic properties time-dependent, such that a material can manifest quite different behavior in response to deformation at different speeds [32–34]. Biological forces can be divided into two timescales, a short timescale where cells sense the mechanical properties of their surroundings by relatively rapid protrusion and retraction of filopodia [32–34]. In this state, the matrix may appear as a significantly stiff material [32–34]. In larger-scale structures, the incorporation of contractile cell types may lead to the presence of contractile forces that operate continuously over a long period of time. Under such conditions, the matrix may be unable to show any resistance to very slow deformation [32–34].

The critical role of the matrix in epithelial cell biology makes organoids an ideal tool to simulate cell-cell interaction *in vitro* as cells can interact freely within an ECM and can simultaneously form contacts with neighboring cells (Hui et al., 2018). In this study, we modeled the effect of normal healthy lung fibroblast and airway smooth muscle (NHLF and NHASM, respectively) stromal cells on the development of healthy primary human bronchial epithelial (NHBE) cells. We hypothesized that interactions between NHBE:NHLF:NHASM would change the morphology of epithelial organoid spheroids and aimed to investigate whether coculture in organoids could mimic a human airway.

## Results

### Branched tubular structures formed by fibroblast (NHLF) and epithelial cell (NHBE) cocultures

**Figure 1:**
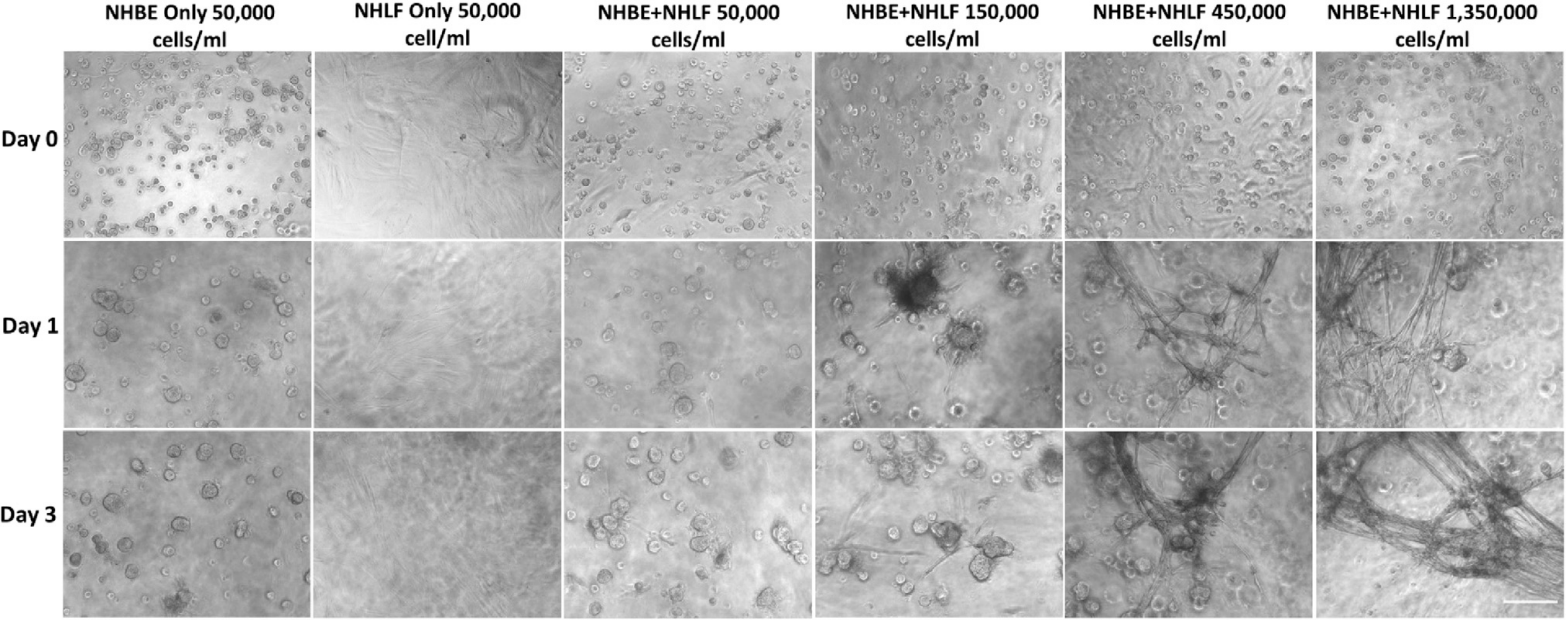
Normal healthy human bronchial epithelial (NHBE) cell and normal healthy human lung fibroblast (NHLF) coculture NHLF seeding density was increased from 6,000 to 1,350,000 cells/ml and NHLF cells allowed to adhere to the bottom of wells of a 96 well plate before being layered with 25% Matrigel and then seeding NHBE cells in 5% Matrigel. Increases in NHLF resulted in increased numbers of tubular structures formed by epithelial cells. Images are representative of those from 2 wells from each experiment and n = 3 biological repeats. Scale bar = 100μm.

**Figure 2:**
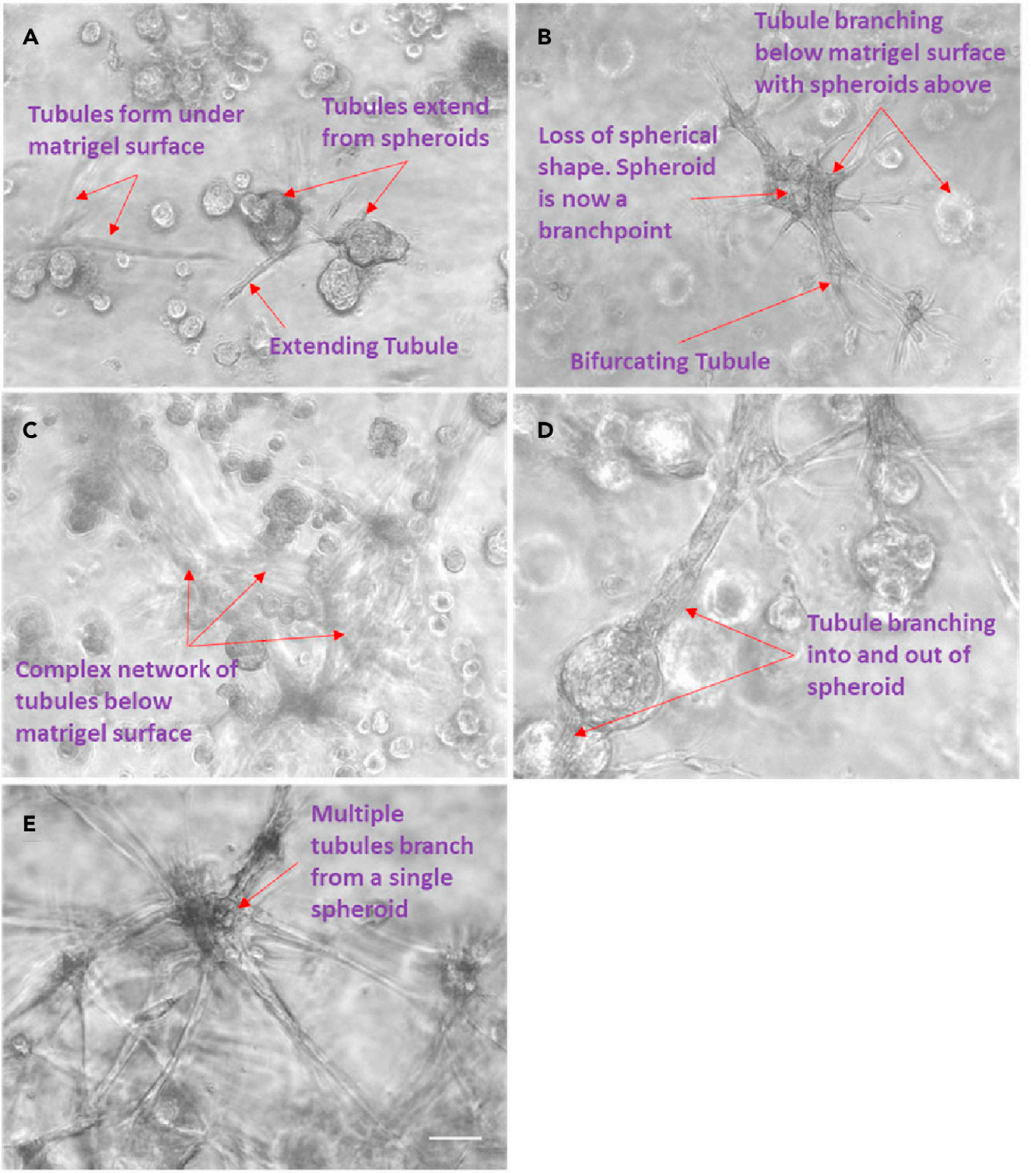
Bronchotubule formation (A–E) Normal healthy human bronchial epithelial (NHBE) cells first aggregated into spheroids (A) that grew long rod like protrusions (B) that eventually formed into an interconnected set of tubular structures that looked ganglionic (C and E). Extending tubules proximally formed a bulbous structure that branched (D). Images are representative of those seen across n = 3 donors. Scale bar = 100 μm.

We confirmed previous reports of the formation of spheroidal lumen-containing structures (Tan et al., 2017; Barkauskas et al., 2017) (Figure 1) and investigated how incorporating fibroblasts into airways epithelial cell culture influences morphology. NHLF and NHBE were first cocultured. NHLF were seeded as a monolayer and overlayed with 25% Matrigel for 24 h after which NHBE in a 5% Matrigel layer were added. At low NHLF levels (<150,000 cell/ml), NHBE aggregated into spheroidal structures after 0–3 days of culture being similar to those observed in NHBE-only controls as described previously (Figure 1). However, at higher NHLF numbers (>150,000 cell/ml), NHBEs formed rod-like structures that connected distinct epithelial spheres at around 3 days (Figures 1 and 2).

**Figure 3:**
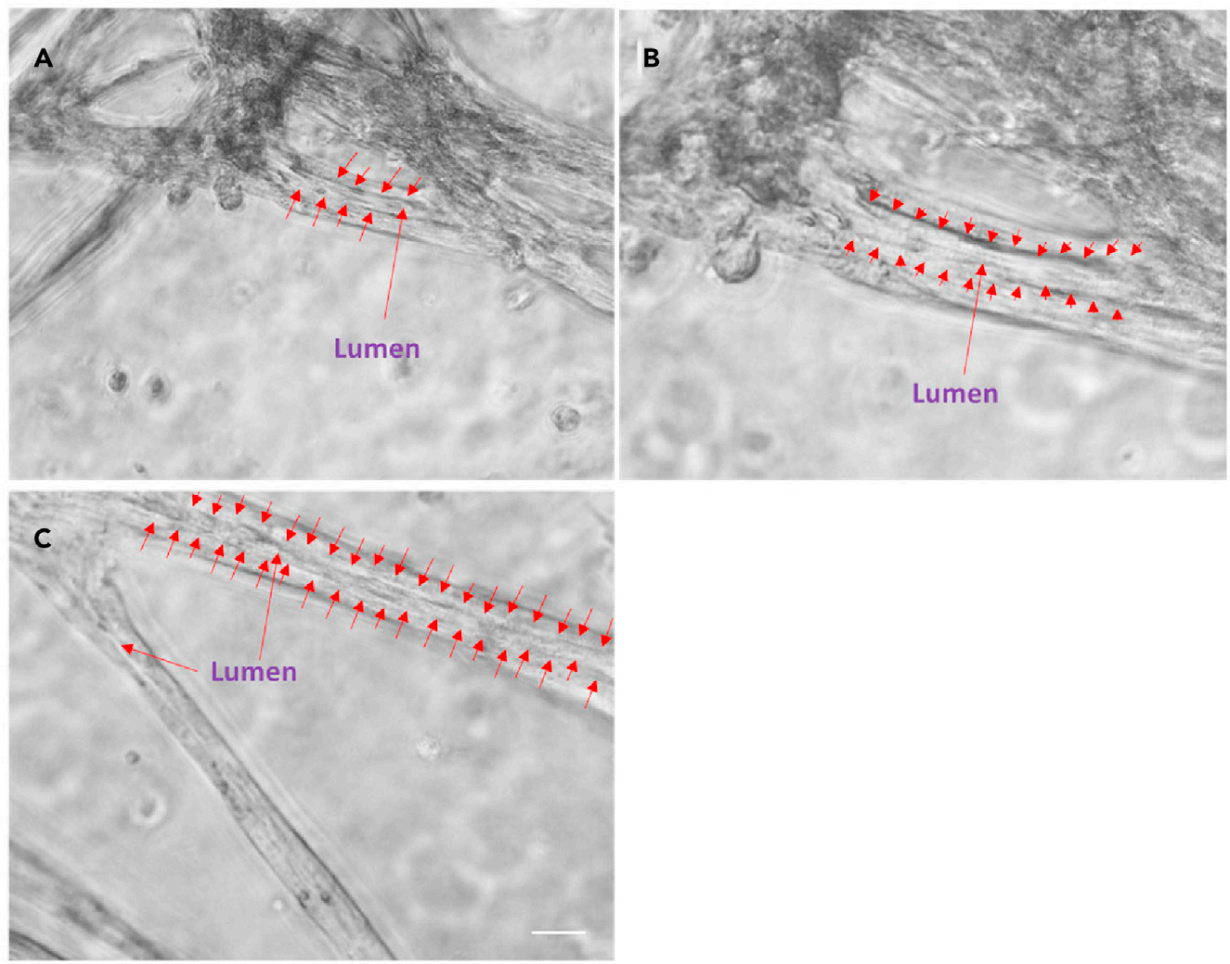
Bronchotubule morphology (A–C) Rod like structures formed lumens by Day 3 (red arrows A–C). Results are representative of bronchotubule formation in 2 wells per plate and in n = 3 biological repeats. Scale bar = 100 μm.

Regular feeding was required as the media became acidic after 12 h reflecting the rapid growth of cells under these conditions. Within 3 days, the rods merged together and formed lumina (Figures 1, 2, and 3). We have termed these previously unreported structures *bronchotubules*. Further increasing the NHLF concentration to 450,000–1,350,000 cell/ml shortened the time to rod formation to 24 h (Figure 1). Interestingly the terminal points of the growing bronchotubules formed rounded bulb structures (Figure 1, Figure 2, Figure 3). The culture survived for 4–6 days; however, at this time-point, tubule structures collapsed.

### Normal human smooth muscle cells (NHASM) incorporate realistically into bronchotubules but do not rescue tubule collapse

**Figure 4:**
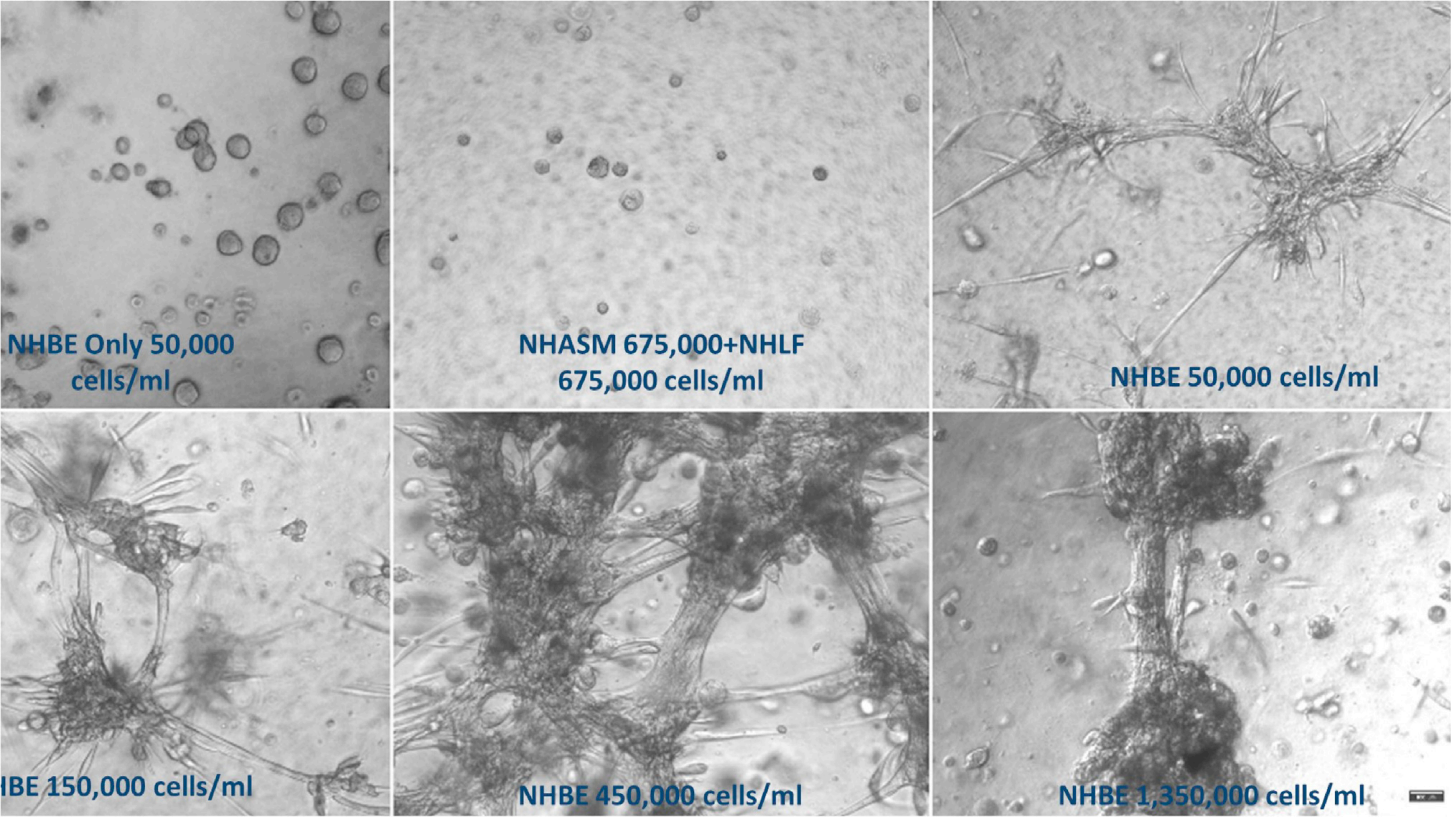
Epithelial cell (NHBE) concentration optimization (A–F) NHBE concentration was varied while fibroblast (NHLF) and smooth muscle (NHASM) seeding density was kept constant at 675,000 cells/ml (A–F). 10× magnification, scale bar = 100 μm. Images are representative of those seen across n = 3 donors.

Bronchiole physiology contains airway smooth muscle that bands around the tube providing structural support and control luminal dimensions. To model this micro architecture, NHASM and NHLF cells were added to the culture to stabilize the tubules (Figure 4). The stromal cell concentrations were kept constant at 1,350,000 cell/ml using equal numbers of both NHLF (675,000 cell/ml) and NHASM (675,000 cell/ml) cells. In control experiments, NHBE cells alone (Figure 4A) and NHLF and NHASM in the absence of epithelial cells, stromal cultures did not branch (Figure 4B). However, in the triple culture, bronchotubules were once again observed and wells with the highest NHBE levels (1,350,000) resulted in the thickest tubes (200–500 μm) (Figures 4C–4F).

To demonstrate the incorporation of smooth muscle functionality into the bronchotubules, we stimulated the bronchotubules with the muscarinic receptor antagonist carbochol (10^−3^M). In a pilot experiment, this caused the tubules to contract (Figure S1), suggesting that the NHASM are incorporated into the tubules. The triple-culture system incorporated smooth muscle and may recapitulate the contractile function of natural airway but this needs to be confirmed. However, the bronchotubules were not long-lived and collapsed between 4 and 6 days as with the simpler NHBE-NHLF co-cultures.

### Developing a mechanically controlled scaffold for long-term stabilization of tubules

For effective application as an organoid, it is essential to stabilize the bronchotubule structures for longer-term culture, beyond the initially obtained 4–6 days. Such longer-term stability is also inherently more representative of physiological conditions. With this in mind, we hypothesize that the tubule collapse at 4–6 days was mechanically driven. Since the coculture incorporates significant numbers of contractile cells (fibroblasts and smooth muscle), there is a natural tendency for cell-contraction to shorten and for tubules to collapse. This tendency must also exist for airway tubules *in vivo* and is prevented by the mechanical integrity of the surrounding ECM. Since 25% Matrigel has shown itself suitable to stimulate bronchotubule formation, we took this as a starting point, looking to modify its mechanical properties without altering the biochemistry of the material.

**Figure 5:**
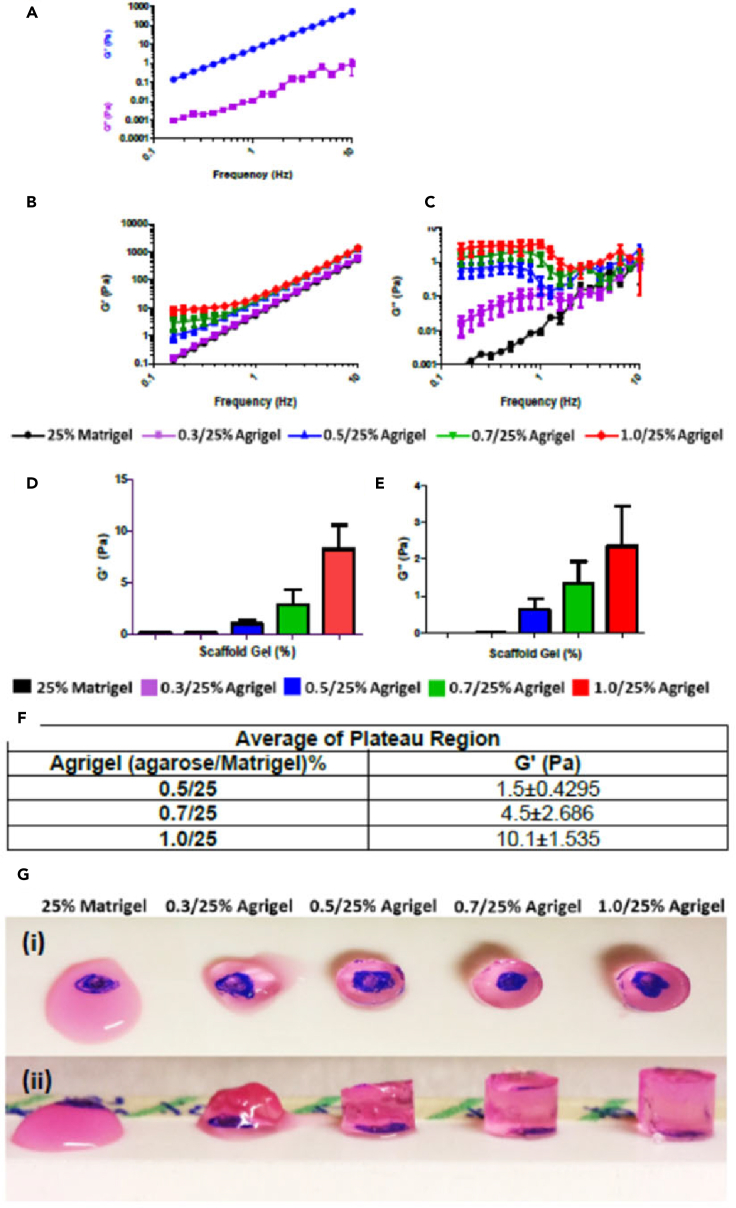
Viscoelastic properties of agrigel scaffold (A–E) Storage modulus (G′) and loss modulus G” of 25% Matrigel alone (B) Storage modulus and (C) loss modulus of agrigel at different concentrations (D). Storage modulus (G′) and (E) loss modulus (G″) of agrigel at different concentrations measured at 0.16Hz. Results are presented as mean ± SEM of n = 3 independent experiments. (F) Average G′ of plateau region showing increasing scaffold stiffness with increasing agarose concentration. (G (i)) Clarity of agrigel scaffold blue dot below gel can be clearly visualized. (G (ii)) Stiffness of agrigel increases with increasing agarose concentration.

The mechanical properties of 25% Matrigel were first measured (Figure 5A). It can be clearly seen that the storage modulus, *G′,* quantifying elastic-solid-like properties, is substantially greater than the loss modulus, *G″*, quantifying viscous-liquid-like properties, for all frequencies measured. Hence, 25% Matrigel can be broadly characterized as a solid-like elastic material, at least within the measured range. However, the time-dependence of *G′* follows a power-law behavior with frequency, such that at lower frequencies, its value is still decreasing with no sign of plateauing at any minimum value. Physically, this implies that 25% Matrigel shows substantial stiffness when it is rapidly deformed; however, for slower, longer-term deformations, the elastic resistance to deformation falls with no lower limit, meaning that the material will be unable to resist a constant continuously applied force.

To modify the 25% Matrigel without altering the concentration of its biochemical factors, we incorporated agarose as a stiffening reagent. Agarose is a good candidate since it is biocompatible but not known to bind cell surface receptors, so that its influence on the tissue development should be purely mechanical. Furthermore, agarose gels set solid with decreasing temperature and do not require chemical cross-linking agents in contrast to other stiffening agents such as alginate, which sets after addition of calcium. The mechanical properties of agarose-25% Matrigel mixed gels, which we term *“agrigels”* were measured using parallel-plate rheometry (Figures 5B and 5C). The agrigels show, *G’ > G’’* at all frequencies, although the gap between the storage and loss moduli narrows at low frequency where these must be considered truly viscoelastic materials. However, the low frequency behavior of the storage modulus, *G′*, is very different from pure Matrigel (Figure 5B). Rather than continuing to decrease as a power law with decreasing frequency (straight line on plot), G′ deviates upwards, approaching a plateau value (Figure 5B). This plateau is clearly seen for 0.7% agarose and is a reasonable extrapolation for 0.5% and 0.3%. The presence of a low-frequency plateau is highly significant, implying that the material will be able to support a long-term constant force and thus could provide a viable scaffold for large-scale contractile tissues such as the bronchotubule system (Figure 5B). The value of stiffness (*G′*) was controlled by the quantity of added agarose: the value of *G′* in the plateau region increased by up to 10,000-fold compared with pure 25% Matrigel with agarose concentrations from 0.3 to 1.0% (Figures 5D–5F). Intriguingly this was a much greater range of stiffnesses than was seen using pure agarose gels at the same concentrations (Figure S2). This ability to resist long-term forces was qualitatively confirmed by the ability of agrigel constructs to retain their shape under gravity better than pure 25% Matrigel (Figure 5G).

We thus consider the agrigel a well-suited unique scaffold system for 3D culture of complex-structured contractile organoids, demonstrating the key ability to support low-frequency/long-time forces and with stiffness control across four orders of magnitude without changing the underpinning biochemistry of the matrix.

### The increased stiffness of the agrigel scaffold enables long-term culture of bronchotubules without collapse

**Figure 6:**
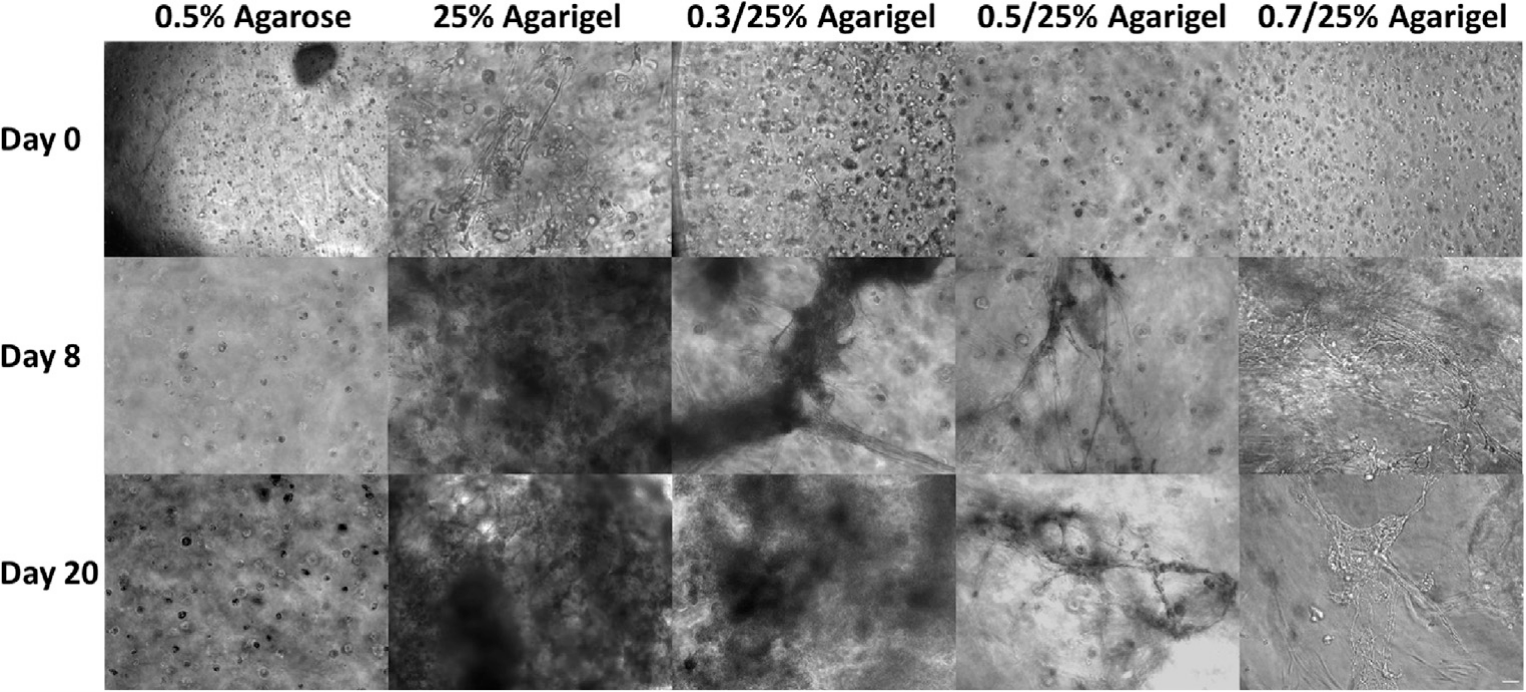
Scaffold optimization Triple culture of normal healthy human bronchial epithelial cells (NHBE), normal healthy human lung fibroblasts (NHLF) and normal healthy human airway smooth muscle (NHASM) cells in different scaffolds. Cell seeding density; NHBE = 450,000 cells/ml, NHLF = 675,000 cells/ml and NHASM = 675,000 cells/ml. 10× magnification, scale bar = 100 μm. Images are representative of n = 3 using cells from the same patient.

To assess whether matrix stiffness affected the durability of organoid cultures, we examined whether addition of agarose to enhance Matrigel viscosity had an impact on bronchotubule formation and sustainability. Bronchotubules were grown in 0.3%, 0.5%, and 0.7% agrigel, with 0.5% agrigel revealing itself as an optimal culture environment for this system. The tubules cultured in 0.3% agarose containing agrigel behaved similarly to 25% Matrigel and collapsed by day 8. In 0.7% agarose containing agrigel, tubules looked thinner compared to that of 0.5% agrigel (Figure 6) and were unable to form a complex network, with spheroidal structures the predominant morphology. Contrastingly, in 0.5% agrigel, bronchotubules successfully formed after 8 days and survived intact until the end of the experiment at 20 days.

**Figure 7:**
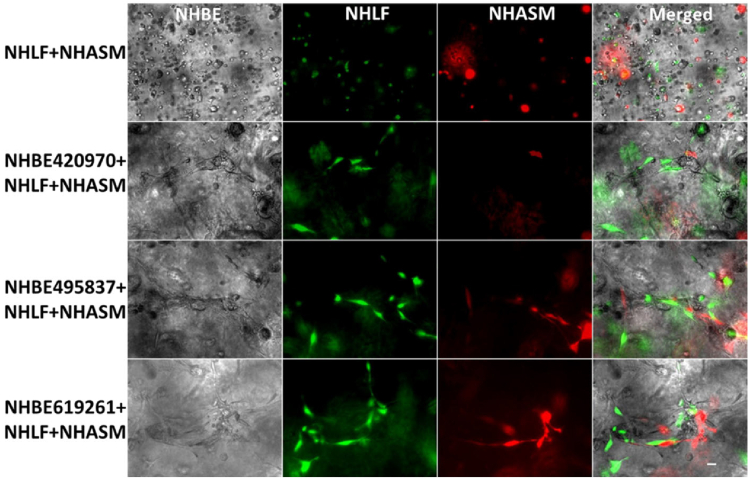
Organization of culture-generated tissue: Bronchotubule Triple Culture in 0.5/25% Agrigel Triple culture of lentivirally transduced of normal healthy human lung fibroblasts (NHLF, green) and normal healthy human airway smooth muscle (NHASM, red) cells at 675,000 cells/ml respectively with normal healthy human bronchial epithelial cells (NHBE) at 450,000 cells/ml. NHLF and NHASM do not form tubules in agrigel compared to triple culture which form tubules over 20 days cultures. Magnification 10×, Scale bar is 100 μm. Images are representative of those from n = 3 different donors.

Based on these results and that of the rheological analysis (Figures S3 and S4), we selected agrigel containing 0.5% agarose (0.5% agrigel) as providing an appropriate balance between the stiffness necessary to support the long-term contractile forces while also delivering a sufficiently compliant gel to allow for cell-migration and tubule growth. Bronchotubules grown in 0.5% agrigel resembled morphologically the airway *in* vivo. In contrast, cells grown in 0.5% agarose alone were unable to form tubular or spheroidal structures indicating the critical role of the Matrigel (Figure 7). These data show that the mechanical properties of the matrix influence tubule formation with the bifurcating tubules growing unidirectionally rather than undertaking nonspecific branching in all directions. All further experiments were performed using 0.5% agrigel.

**Figure 8:**
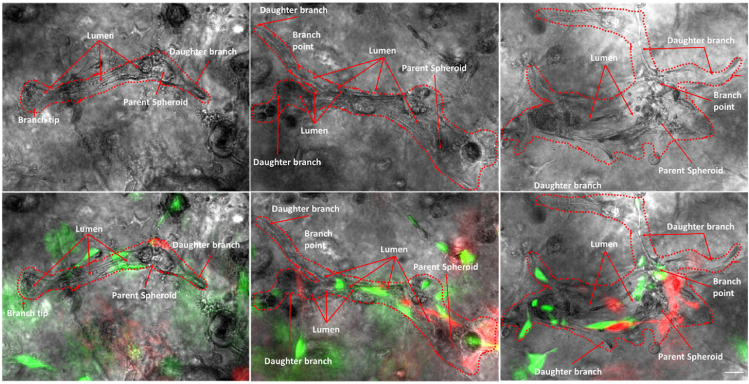
Organization of culture-generated tissue: Bronchotubule Triple Culture in 0.5/25% Agrigel Day 20 Bronchotubules arise from spheroids and form bifurcating branches over 20 days culture. Normal healthy human lung fibroblasts (NHLF, green) and normal healthy human airway smooth muscle (HASM, red) cells surround and support the growth of normal healthy human bronchial epithelial cells (NHBEC, gray). Images are representative of n = 3 experiments using NHBECs from 3 different donors. Scale bar = 100 μm.

Next, we determined whether culture with different cell types formed the correct airway architecture. We expected that NHBE would be surrounded by NHLF and that NHASM cells would band across the outer edge of the bronchotubule. To elucidate the position of NHLF and NHASM in bronchotubule structure in 0.5/25% agrigel, NHLF and NHASM were labeled with lentiviral-transfected yellow fluorescence protein and mCherry, respectively (Figure S5). The culture was repeated with primary NHBE from 3 healthy donors. NHBE cells (gray) were surrounded by NHLF (green) with NHASM cells (red) forming the outer edge of the tubules mimicking the human airway and indicating that epithelial-stromal stratification occurs in this model (Figures 7 and 8). Tubules from each NHBE cell donor were maintained for 20 days although cells from each patient formed morphologically different bifurcating fractal structures where bronchotubules from each patient-generated daughter branches of different size, length, and shape (Figure 8). Epithelial cells that did not migrate into the gel and interact with other stromal cells formed spheroids but not bronchotubules (Figure S6).

These results show that bronchotubule culture is able to recapitulate the airway cellular architecture and that distinct cell lineages are autonomously able to migrate and organize themselves into expected regions within the developing bronchotubule even though they originate from three separate healthy donors. The presence of a cellular lumen indicates that epithelial cells may line the inner lumen as only stromal cells could be observed outside of the tubules and maybe undergoing apical-basal polarization observed in luminogenesis. These data need to be confirmed by further experiments. However, bronchotubule formation is not only cell-dependent but requires an ECM with the right level of viscoelasticity. We have shown that ECM stiffness is crucial in patterning and holding the structure (Figures 7 and 8) which collapsed after 4 days in Matrigel alone. These experiments also suggest that primary human airway cells are inherently programmed to form the correct airway patterning when exposed to the right ECM mechanical niche.

## Discussion

We report here for the first time, the generation of functional contracting lung tubular branching NHBEC-derived organoids in the presence of stromal NHLF and NHASM cells, that we have termed bronchotubules. We introduced a new scaffold material, agrigel, whose time-dependent mechanical properties were designed to allow long-term culture of these contractile organoids. To our knowledge, agarose in combination with Matrigel has not previously been used as a mixed gel scaffold for organoid culture, although applications of collagen-agarose mixed gels are reported (Shin et al., 2016; Kopf et al., 2016; Ulrich et al., 2010). Bronchotubule organoids developed in this study, display epithelial-stromal stratification and cannot form without epithelial-stromal signaling.

Previous studies have reported that mixed stromal–epithelial cell populations can form spheroidal structures (Danahay et al., 2015) that branch and lose their spheroidal identity. Tubules formed in 25% and 0.3% (Figures 4 and 6) Matrigel merge into each other as seen in our bronchosphere culture after 4 days whereas tubules cultured in the stiffer 0.5% agrigel continue branching in a unidirectional manner with adjacent tubules not merging into each other (Figures 6, 7, and 8). However, cells cultured in only 0.5% agarose were not able to branch showing that Matrigel itself is needed for tubule formation. Furthermore, tubules grown in a gel that is stiff but not compliant enough such as the 0.7% agrigel may not allow for enough migration and tubule growth resulting in thinner tubules (Figure 6). These data together show that the interplay between scaffold stiffness and compliance together with ECM factors are paramount for tissue patterning. Bronchotubules grown in 0.5% agrigel do not appear to reach quiescence and continue to branch and grow for up to 3 weeks – the maximal duration of analysis.

During lung development *in vivo*, branching ceases when tip cells begin to form alveolar structures with epithelial cells acquiring a SOX9/ID2+ identity and eventually forming ATI and ATII epithelial cells (Jones et al., 2018; Rockich et al., 2013; Yuan et al., 2018). This SOX9/ID2+ phenotype is theorized to arise from tip cells of nascent bifurcating tubules. Our study did not include alveolar populations and perhaps the addition of ATII cell populations are required to give a stop signal to branching and enable the formation of alveolar structures (Rockich et al., 2013; Yuan et al., 2018; Barkauskas et al., 2017; Hogan et al., 2014). The formation of tubules may indicate that epithelial cells are preprogrammed to create tubular structures in certain microenvironments.

Organoid culture methods have applied various matrix scaffolds such as Matrigel or collagen gels (Barkauskas et al., 2017; Mcqualter et al., 2010; Chen et al., 2012; Danahay et al., 2015). Furthermore, the concentration of scaffold gel used varies in the literature, which may have major effects on the resultant organoids formed. We have shown that even a 0.2% change in agarose concentration has a profound effect on the type of structures that form. Varying this quantity may unwittingly increase or decrease exogenous proteins that stimulate organoid assembly. Maybe the level of resistance of very stiff 0.7% gel to cellular migration was too high, resulting in less tubular and more spheroidal structures. Studies where epithelial cells grown in 50% Matrigel results in tracheosphere organoids that are clonal arising from the division of a single progenitor cell (Rock et al., 2009) contrary to 25% Matrigel formulations where organoids develop by the migration and aggregation of cells. The role of cellular migration and rearrangements *in vivo* are crucial for lung branching morphogenesis during development. For example, Bangasser et al have shown that stromal progenitor migration to the tips of nascent lung tubes and their differentiation into smooth muscle cells is crucial for tubular bifurcation (Kim et al., 2015). This requires the surrounding matrix to be compliant and viscous enough to allow cells to pass indicating that the matrix plays a major role in cellular migration and that there is an optimal fitness for cellular ability to migrate through matrix (Bangasser et al., 2017). Whilst comparing organoid behavior across studies is difficult, it is evident that the matrix scaffold used is important.

In summary, this method has enabled long-term stable culture of the present bronchotubule system. Since the mechanical microenvironment can easily be varied by specifically increasing or decreasing the agarose concentration, it will be possible to mimic different scaffold environments of different airways diseases like COPD and IPF. The bronchotubule system opens the possibility to investigate questions around the role of cell–matrix interaction and cell–cell communication in tissue patterning, repair, and disease *in vitro*. We anticipate the use of hybrid cultures to investigate the interactions between diseased and healthy cells. Further advancement of organoid models like these will expand our knowledge of how cell–cell or cell–matrix interactions drive the development and repair of lung tissue.

### Study limitations

This study has several limitations including the sole use of structural cells from healthy subjects and exclusion of immune cells and the lack of confocal imaging to confirm tubule structures formed by NHBE:NHASM:NHLF cocultures. Culturing bronchotubules using diseased lung cells such as COPD-HBEC may generate aberrantly branching tubules due to dysregulated cell–cell signaling. If cells are preprogrammed to make these structures, diseased lineages should recapitulate abnormal development. Furthermore, disease–healthy cell hybrid bronchotubules such as NHBE with ASMs and COPD fibroblasts or COPD HBECs with NHLF and NHASM cells could help elucidate disease driving cell lineages of abnormal developmental processes and whether one could rescue bronchotubule phenotype by replacing the disease-driving lineage with a healthy lineage. Furthermore, a recent study showed that healthy cells grown in matrix from diseased patients result in organoids that recapitulate diseased lung architecture (Hedstrom et al., 2018). Future studies should examine the differentiation of epithelial cell subtypes in bronchotubule cultures over time using single-cell RNA-sequencing (Vieira Braga et al., 2019) comparing the data with bronchial biopsies from healthy and COPD donors.

## STAR★Methods

### Key resource table

**Table:** 

REAGENT or RESOURCE	SOURCE	IDENTIFIER
**Bacterial and virus strains**		

pMD.2G	Gifted from Crick Institute	N/a
psPAX2	Gifted from Crick Institute	N/a
Yellow Fluorescence Protein (YFP) Plasmid transfected bacteria	Adgene	Cat. #45295
mCherry Plasmid transfected bacteria	Adgene	Cat. #36084

**Biological samples**		

NHBE	Lonza	Lot#0000420970
NHBE	Lonza	Lot#0000495837
NHBE	Lonza	Lot#0000619261
NHLF	Promocell	Lot#101901.2
NHASM	Gifted by Dr. Charis Charalambous	N/a

**Chemicals, peptides, and recombinant proteins**		

Bronchial Epithelium Basal Medium (BEBM)	Lonza	Cat. #CC-3170
Dulbecco’s Modified Eagle’s Media (DMEM) High Glucose	Gibco,	Cat. #10741574
EGF	Lonza	Cat. #CC-4175
Epinephrine	Lonza	Cat. #CC-4175
Hydrocortisone	Lonza	Cat. #CC-4175
Insulin	Lonza	Cat. #CC-4175
Transferrin	Lonza	Cat. #CC-4175
All-Trans Retinoic Acid (RA)	Sigma-Aldrich	Cat. #R2625-50MG
Trypsin/EDTA	Lonza	Cat. #: CC-5034
Trypsin Neutralising Solution (TNS)	Lonza	Cat. #: CC-5034
Matrigel	Corning	Cat. #354230
Dulbecco’s Modified Eagle’s Media (DMEM)	Sigma-Aldrich	Cat. #11960069
L-Glutamine (200mM)	Sigma-Aldrich	Cat. #G7513
Penicillin/Strptomycin	Sigma-Aldrich	Cat. #P4333-100ML
Lauria Broth	Sigma-Aldrich	Cat. # L3022-1KG
Ampicillin	Invitrogen	Cat. #A5354-10ML
OPTI-MEM	Invitrogen	Cat. # 31985062
Lipofectamine 2000	Invitrogen	Cat. #11668019

**Critical commercial assays**		

Qiagen Maxiprep Kit	Qiagen	Cat. #12162
Lenti-X™ Concentrator kit	Lenti-X™ Concentrator kit	Lenti-X™ Concentrator kit
Takara Bioscience	Takara Bioscience	Takara Bioscience

**Experimental models: cell lines**		

HEK293FT cells	Gifted from Crick institute	N/a

**Other**		

0.45μm polyethersulfone filter	Sigma-Aldrich (Milex)	Cat. #SLHP033RB
6 well cell culture plate 9.5cm^2^/well	Corning	Cat. #3516

### Resource availability

#### Lead contact

Further information and requests for resources and reagents should be directed to and will be fulfilled by the Lead Contact, Prof. Ian M. Adcock (ian.adcock@imperial.ac.uk).

#### Materials availability

Requested materials are available from the Lead Contact.

### Experimental model and subject details

All human NHBE cells were purchased from Lonza Group AG. NHLF cells were purchased from PromoCell. NHASM cells were generously donated by Dr. Charis Charalambous.

#### Cell culture

Never-smoker human airway epithelial cells and culture media from Lonza (Basel, Switzerland) were cultured according to company’s instructions and used at passage 2 (P2). Normal Human Lung Fibroblasts from Promocell (Heidelberg, Germany) were cultured in accordance with the company’s instructions and were used at P4-P5. Normal Human Airway Smooth Muscle cells donated by Dr. Charis Charalambous (NHLI, Imperial College) were cultured in Dulbecco’s Modified Eagles Medium (DMEM, Sigma) supplemented with 10% fetal calf serum (FCS, Sigma, Poole, UK), 5% L-glutamine (200nM, Sigma) and penicillin/streptomycin (0.1 mg/ml, Sigma) and used at P4-P5 as described (Wiegman et al., 2015). All tissue was obtained with appropriate consent and its use approved by the Ethics Committee of the Royal Brompton and Harefeld National Health Service Trust.

**Table:** 

Cell Type	Age	Sex
NHBE	69	F
NHBE	69	F
NHBE	53	M
NHLF	48	F
NHASM	65	M

#### NHBE-NHLF cell co-culture

NHLF were seeded onto 96 well plates at either 25,000, 75,000, 225,000 or 675,000 cells/ml in 0.1ml differentiation media and adhered overnight. Media was removed, and cells were coated with 25% Matrigel in differentiation media containing 100nM RA for 2h at 37°C. NHBE cells were seeded at 50,000 cells/ml in 5% Matrigel in differentiation media supplemented with 100nM RA and fed every 2 days.

#### NHBE-NHLF-NHASM cell triple culture

NHBE, NHLF and NHASM cells were trypsinised and centrifuged at 220 x g and were mixed in 50ml falcon tubes: NHBE 45,000 cells/well and NHLF and NHASM at 67,500 cells/well. Agrigel solution was mixed with cells and pipetted into transwells precoated with 0.5/25% Agrigel. Differentiation medium with 100nM RA was added to basal wells. The culture was incubated at 37°C for 20 days. Media was replenished every 2 days.

### Method details

#### Agrigel formulation

Matrigel was diluted on ice to 25% in differentiation media (1:1 Dulbecco's Modified Eagle's Medium (DMEM, Sigma, Poole, UK) and Bronchial Epithelial Basal Medium (BEBM, Lonza, Slough, UK) with growth factors from the Lonza bullet kit excluding retinoic acid (RA) and triidothyrine) supplemented with 100nM RA). 2% low melting point agarose (Sigma, Poole, UK) was heated to 45°C and added to 25% Matrigel to a final concentration of 0.5% or 0.7% low melting point agarose (Sigma, Poole, UK). Gels were mixed quickly until a uniform pink color was observed forming Agrigel (0.5% or 0.7% low melting point agarose/25% Matrigel).

#### Lentiviral transduction

Yellow fluorescence protein (pLEX_970_puro_DEST_YFP gifted by William Hahn (Addgene plasmid # 45295)) and mCherry (plv_mCherry gifted by Pantelis Tsoulfas, (Addgene plasmid # 36084)) plasmid in transfected bacteria were amplified and extracted with Qiagen Maxiprep kit (Qiagen, Manchester, UK). HEK293FT cells (gifted by Dr. Carlos Lopez Garcia Crick Institute) were transfected with YFP or mCherry, psPax2 and pMD.2G viral RNA plasmid envelope in OPTI-MEM with lipofectamine 2000. Lenti-X™ qRT-PCR Titration Kit (Takara biosciences) was used to calculate the viral multiplicity of infection (MOI) of 3 that was used to infect NHLF and NHASM cells.

#### Microscopy

Organoids were visualized with Leica DMI6000 microscope and analyzed with ImageJ version 1.52e (https://imagej.net/).

#### Mechanical characterization of gels

50μl of either 25% Matrigel, Agarose (0.3, 0.5, 0.7 or 1.0%) or Agrigel (0.3/25, 0.5/25, 0.7/25 or 1.0/25%) solution was instantly poured into a Teflon mold that was 8mm diameter and 5mm in height. Gels were solidified at 37°C/5% CO_2_ for 20 minutes and incubated for 24h in differentiation media with all supplements to mimic the cell-based experimental conditions and rheometry performed using a Rheometer discovery HR1 (TA Instruments using TRIOS software, New Castle, DE, USA). The base plate was heated to 37°C, gels were removed from the Teflon mold, placed on the baseplate and were compressed with a 10mm diameter compressive plate. Storage modulus (G’) and loss modulus (G”) were acquired from oscillatory shear measurements (frequency 0.1-25rad/s).

We used parallel plate oscillatory shear rheometry to measure the time-dependent viscoelastic properties of scaffold candidates. This technique outputs two material properties: *the storage modulus, G’*, which stands for solid-like stiffness, and becomes proportional to Young’s Modulus for a purely solid material, and the *loss modulus, G’’*, which represents liquid-like properties and is closely related to the viscosity for a pure liquid (for a full interpretation see (Hunter, 2001)). Both of these were determined as a function of the deformation frequency to reveal the material’s time-dependent properties.

### Quantification and statistical analysis

A Mann-Whitney U test was used for analyses between two non-normally distributed groups. One-way ANOVA Kruskal-Wallis test was used to assess statistically significant differences between means of two non normally distributed groups. All statistical analyses were performed in GraphPad Prism 6.0 software.

## Data Availability

All relevant data are included in the present article and its supplementary information or are available from the Lead Contact upon request. The study does not contain custom code. Any additional information required to reanalyze the data reported in this work paper is available from the Lead Contact upon request.

## References

[bib1] Bangasser B.L., Shamsan G.A., Chan C.E., Opoku K.N., Tuzel E., Schlichtmann B.W., Kasim J.A., Fuller B.J., Mccullough B.R., Rosenfeld S.S., Odde D.J. (2017). Shifting the optimal stiffness for cell migration. Nat. Commun..

[bib2] Barkauskas C.E., Chung M.I., Fioret B., Gao X., Katsura H., Hogan B.L. (2017). Lung organoids: current uses and future promise. Development.

[bib3] Barkauskas C.E., Cronce M.J., Rackley C.R., Bowie E.J., Keene D.R., Stripp B.R., Randell S.H., Noble P.W., Hogan B.L. (2013). Type 2 alveolar cells are stem cells in adult lung. J. Clin. Invest.

[bib4] Barker N., Huch M., Kujala P., van de Wetering M., Snippert H.J., van Es J.H., Sato T., Stange D.E., Begthel H., van den Born M. (2010). Lgr5(+ve) stem cells drive self-renewal in the stomach and build long-lived gastric units in vitro. Cell Stem Cell.

[bib5] Benam K.H., Villenave R., Lucchesi C., Varone A., Hubeau C., Lee H.H., Alves S.E., Salmon M., Ferrante T.C., Weaver J.C. (2016). Small airway-on-a-chip enables analysis of human lung inflammation and drug responses in vitro. Nat. Methods.

[bib6] Bitterman P. (2018). Fibroblast–matrix cross-talk in idiopathic pulmonary fibrosis: cross-links at the crossroads. Am. J. Respir. Cell Mol. Biol..

[bib7] Bonnans C., Chou J., Werb Z. (2014). Remodelling the extracellular matrix in development and disease. Nat. Rev. Mol. Cell Biol.

[bib8] Broutier L., Mastrogiovanni G., Verstegen M.M., Francies H.E., Gavarro L.M., Bradshaw C.R., Allen G.E., Arnes-Benito R., Sidorova O., Gaspersz M.P. (2017). Human primary liver cancer-derived organoid cultures for disease modeling and drug screening. Nat. Med..

[bib10] Chen H., Matsumoto K., Brockway B.L., Rackley C.R., Liang J., Lee J.H., Jiang D., Noble P.W., Randell S.H., Kim C.F., Stripp B.R. (2012). Airway epithelial progenitors are region specific and show differential responses to bleomycin-induced lung injury. Stem Cells.

[bib9] Burgstaller G., Oehrle B., Gerckens M., White E.S., Schiller H.B., Eickelberg O. (2017). The instructive extracellular matrix of the lung: basic composition and alterations in chronic lung disease. Eur. Respir. J..

[bib11] Chen Y.W., Huang S.X., de Carvalho A., Ho S.H., Islam M.N., Volpi S., Notarangelo L.D., Ciancanelli M., Casanova J.L., Bhattacharya J. (2017). A three-dimensional model of human lung development and disease from pluripotent stem cells. Nat. Cell Biol.

[bib12] Danahay H., Pessotti A.D., Coote J., Montgomery B.E., Xia D., Wilson A., Yang H., Wang Z., Bevan L., Thomas C. (2015). Notch2 is required for inflammatory cytokine-driven goblet cell metaplasia in the lung. Cell Rep.

[bib13] Doeing D.C., Solway J. (2013). Airway smooth muscle in the pathophysiology and treatment of asthma. J. Appl. Physiol. (1985).

[bib14] El Agha E., Kheirollahi V., Moiseenko A., Seeger W., Bellusci S. (2017). Ex vivo analysis of the contribution of FGF10(+) cells to airway smooth muscle cell formation during early lung development. Dev. Dyn..

[bib15] Gkatzis K., Taghizadeh S., Huh D., Stainier D.Y.R., Bellusci S. (2018). Use of three-dimensional organoids and lung-on-a-chip methods to study lung development, regeneration and disease. Eur. Respir. J..

[bib16] Hedstrom U., Hallgren O., Oberg L., Demicco A., Vaarala O., Westergren-Thorsson G., Zhou X. (2018). Bronchial extracellular matrix from COPD patients induces altered gene expression in repopulated primary human bronchial epithelial cells. Sci. Rep..

[bib17] Hindley C.J., Cordero-Espinoza L., Huch M. (2016). Organoids from adult liver and pancreas: stem cell biology and biomedical utility. Dev. Biol..

[bib18] Hogan B.L., Barkauskas C.E., Chapman H.A., Epstein J.A., Jain R., Hsia C.C., Niklason L., Calle E., Le A., Randell S.H. (2014). Repair and regeneration of the respiratory system: complexity, plasticity, and mechanisms of lung stem cell function. Cell Stem Cell.

[bib19] Huh D., Matthews B.D., Mammoto A., Montoya-Zavala M., Hsin H.Y., Ingber D.E. (2010). Reconstituting organ-level lung functions on a chip. Science.

[bib20] Hui K.P.Y., Ching R.H.H., Chan S.K.H., Nicholls J.M., Sachs N., Clevers H., Peiris J.S.M., Chan M.C.W. (2018). Tropism, replication competence, and innate immune responses of influenza virus: an analysis of human airway organoids and ex-vivo bronchus cultures. Lancet Respir. Med..

[bib21] Hunter R.J. (2001).

[bib22] Jones M.R., Dilai S., Lingampally A., Chao C.M., Danopoulos S., Carraro G., Mukhametshina R., Wilhelm J., Baumgart-Vogt E., Al Alam D. (2018). A comprehensive analysis of fibroblast growth factor receptor 2b signaling on epithelial tip progenitor cells during early mouse lung branching morphogenesis. Front Genet..

[bib23] Kim H.Y., Pang M.F., Varner V.D., Kojima L., Miller E., Radisky D.C., Nelson C.M. (2015). Localized smooth muscle differentiation is essential for epithelial bifurcation during branching morphogenesis of the Mammalian lung. Dev. Cell.

[bib24] Kopf M., Campos D.F., Blaeser A., Sen K.S., Fischer H. (2016). A tailored three-dimensionally printable agarose-collagen blend allows encapsulation, spreading, and attachment of human umbilical artery smooth muscle cells. Biofabrication.

[bib25] Marinkovic A., Liu F., Tschumperlin D.J. (2013). Matrices of physiologic stiffness potently inactivate idiopathic pulmonary fibrosis fibroblasts. Am. J. Respir. Cell Mol Biol.

[bib26] Mcqualter J.L., Yuen K., Williams B., Bertoncello I. (2010). Evidence of an epithelial stem/progenitor cell hierarchy in the adult mouse lung. Proc. Natl. Acad. Sci. U S A.

[bib27] Michalik M., Wójcik-Pszczoła K., Paw M., Wnuk D., Koczurkiewicz P., Sanak M., Pękala E., Madeja Z. (2018). Fibroblast-to-myofibroblast transition in bronchial asthma. Cell Mol. Life Sci..

[bib28] Miglino N., Roth M., Lardinois D., Sadowski C., Tamm M., Borger P. (2012). Cigarette smoke inhibits lung fibroblast proliferation by translational mechanisms. Eur. Respir. J..

[bib29] Osei E.T., Noordhoek J.A., Hackett T.L., Spanjer A.I., Postma D.S., Timens W., Brandsma C.A., Heijink I.H. (2016). Interleukin-1alpha drives the dysfunctional cross-talk of the airway epithelium and lung fibroblasts in COPD. Eur. Respir. J..

[bib30] Piccolo S., Dupont S., Cordenonsi M. (2014). The biology of YAP/TAZ: hippo signaling and beyond. Physiol. Rev..

[bib31] Rock J.R., Onaitis M.W., Rawlins E.L., Lu Y., Clark C.P., Xue Y., Randell S.H., Hogan B.L. (2009). Basal cells as stem cells of the mouse trachea and human airway epithelium. Proc. Natl. Acad. Sci. U S A.

[bib32] Rock J.R., Randell S.H., Hogan B.L. (2010). Airway basal stem cells: a perspective on their roles in epithelial homeostasis and remodeling. Dis. Model Mech..

[bib33] Rockich B.E., Hrycaj S.M., Shih H.P., Nagy M.S., Ferguson M.A., Kopp J.L., Sander M., Wellik D.M., Spence J.R. (2013). Sox9 plays multiple roles in the lung epithelium during branching morphogenesis. Proc. Natl. Acad. Sci. U S A.

[bib34] Sato T., Stange D.E., Ferrante M., Vries R.G., van Es J.H., van den Brink S., van Houdt W.J., Pronk A., van Gorp J., Siersema P.D., Clevers H. (2011). Long-term expansion of epithelial organoids from human colon, adenoma, adenocarcinoma, and Barrett's epithelium. Gastroenterology.

[bib35] Shin S., Ikram M., Subhan F., Kang H.Y., Lim Y., Lee R., Jin S., Jeong Y.H., Kwak J.-Y., Na Y.-J., Yoon S. (2016). Alginate–marine collagen–agarose composite hydrogels as matrices for biomimetic 3D cell spheroid formation. RSC Adv..

[bib36] Tan Q., Choi K.M., Sicard D., Tschumperlin D.J. (2017). Human airway organoid engineering as a step toward lung regeneration and disease modeling. Biomaterials.

[bib37] Ulrich T.A., Jain A., Tanner K., Mackay J.L., Kumar S. (2010). Probing cellular mechanobiology in three-dimensional culture with collagen-agarose matrices. Biomaterials.

[bib38] Vieira Braga F.A., Kar G., Berg M., Carpaij O.A., Polanski K., Simon L.M., Brouwer S., Gomes T., Hesse L., Jiang J. (2019). A cellular census of human lungs identifies novel cell states in health and in asthma. Nat. Med..

[bib39] Volckaert T., Campbell A., de Langhe S. (2013). c-Myc regulates proliferation and Fgf10 expression in airway smooth muscle after airway epithelial injury in mouse. PLoS ONE.

[bib40] White E.S. (2015). Lung extracellular matrix and fibroblast function. Ann. Am. Thorac. Soc..

[bib41] Wiegman C.H., Michaeloudes C., Haji G., Narang P., Clarke C.J., Russell K.E., Bao W., Pavlidis S., Barnes P.J., Kanerva J. (2015). Oxidative stress-induced mitochondrial dysfunction drives inflammation and airway smooth muscle remodeling in patients with chronic obstructive pulmonary disease. J. Allergy Clin. Immunol..

[bib42] Yuan T., Volckaert T., Chanda D., Thannickal V.J., de Langhe S.P. (2018). Fgf10 signaling in lung development, homeostasis, disease, and repair after injury. Front Genet..

[bib43] Zhou Y., Horowitz J.C., Naba A., Ambalavanan N., Atabai K., Balestrini J., Bitterman P.B., Corley R.A., Ding B.S., Engler A.J. (2018). Extracellular matrix in lung development, homeostasis and disease. Matrix Biol..

