# Supplementary material for: Epithelial–stromal cell interactions and extracellular matrix mechanics drive the formation of airway-mimetic tubular morphology in lung organoids
Source: iScience. 2021 Aug 30;24(9):103061. doi: 10.1016/j.isci.2021.103061 (PMC8450245; doi:10.1016/j.isci.2021.103061)
Supplement: Document S1. Figures S1–S6 [file mmc1.pdf]

iScience, Volume *24*

Supplemental information

Epithelial–stromal cell interactions and extracellular matrix mechanics drive the formation of airway-mimetic tubular morphology in lung organoids

Tankut.G. Güney, Alfonso Muinelo Herranz, Sharon Mumby, Iain E. Dunlop, and Ian M. Adcock

Supplementary Figures

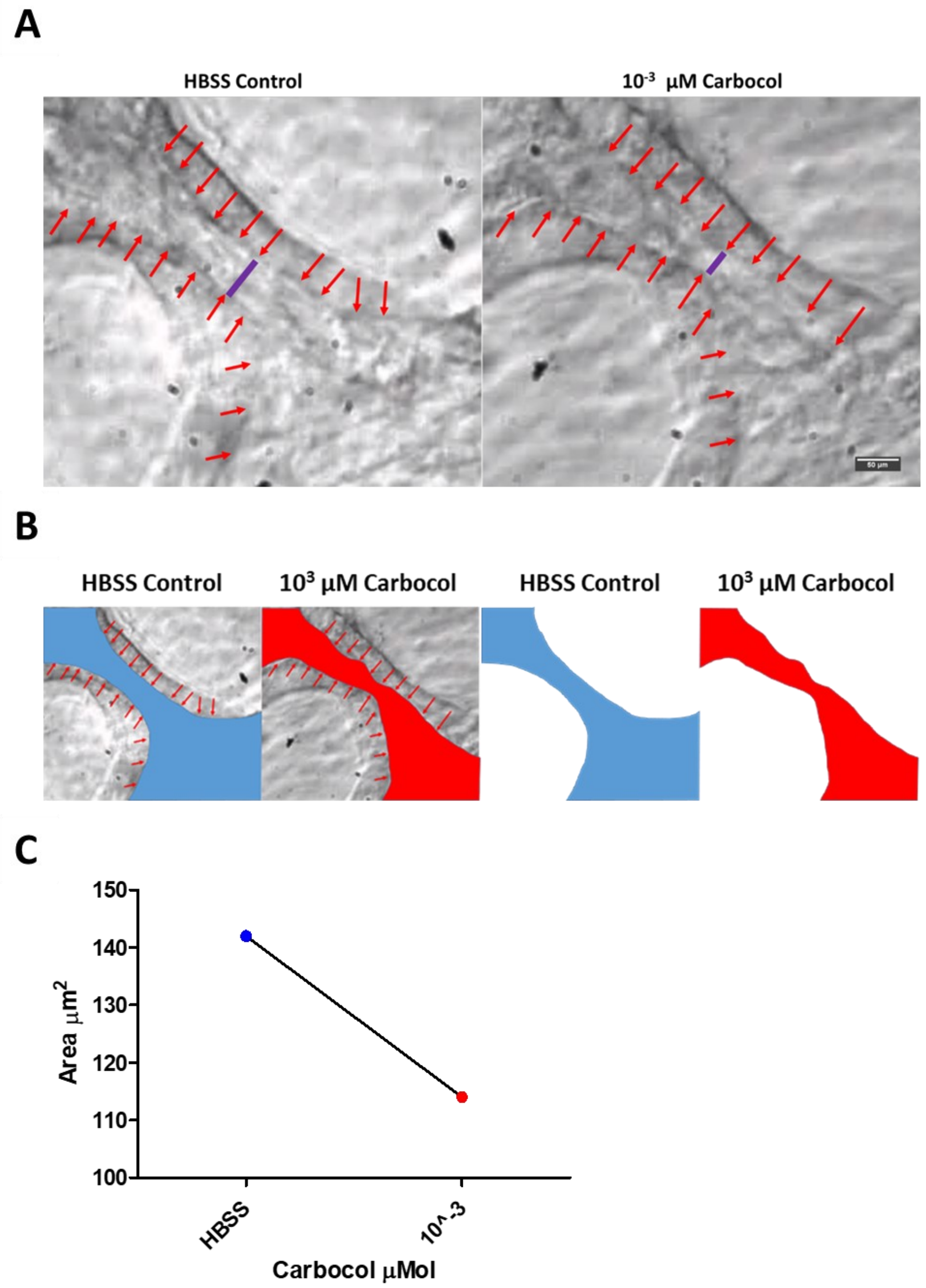

Figure S1. Normal healthy human airway smooth muscle (NHASM) Contraction assay, Related to Figure 4: The functionality of NHASM cells was measured by the addition of the muscarinic agonist carbachol (10^{-3}M) which causes ASM contraction and airway constriction compared to Hank's Balanced Salt Solution (HBSS) buffer control. Purple bar shows luminal contraction, A, area of lumen was measured using imageJ by marking the inside lumen (blue and red), B and C. 40x magnification, Scale bar scalebar= 100µm. n=1.

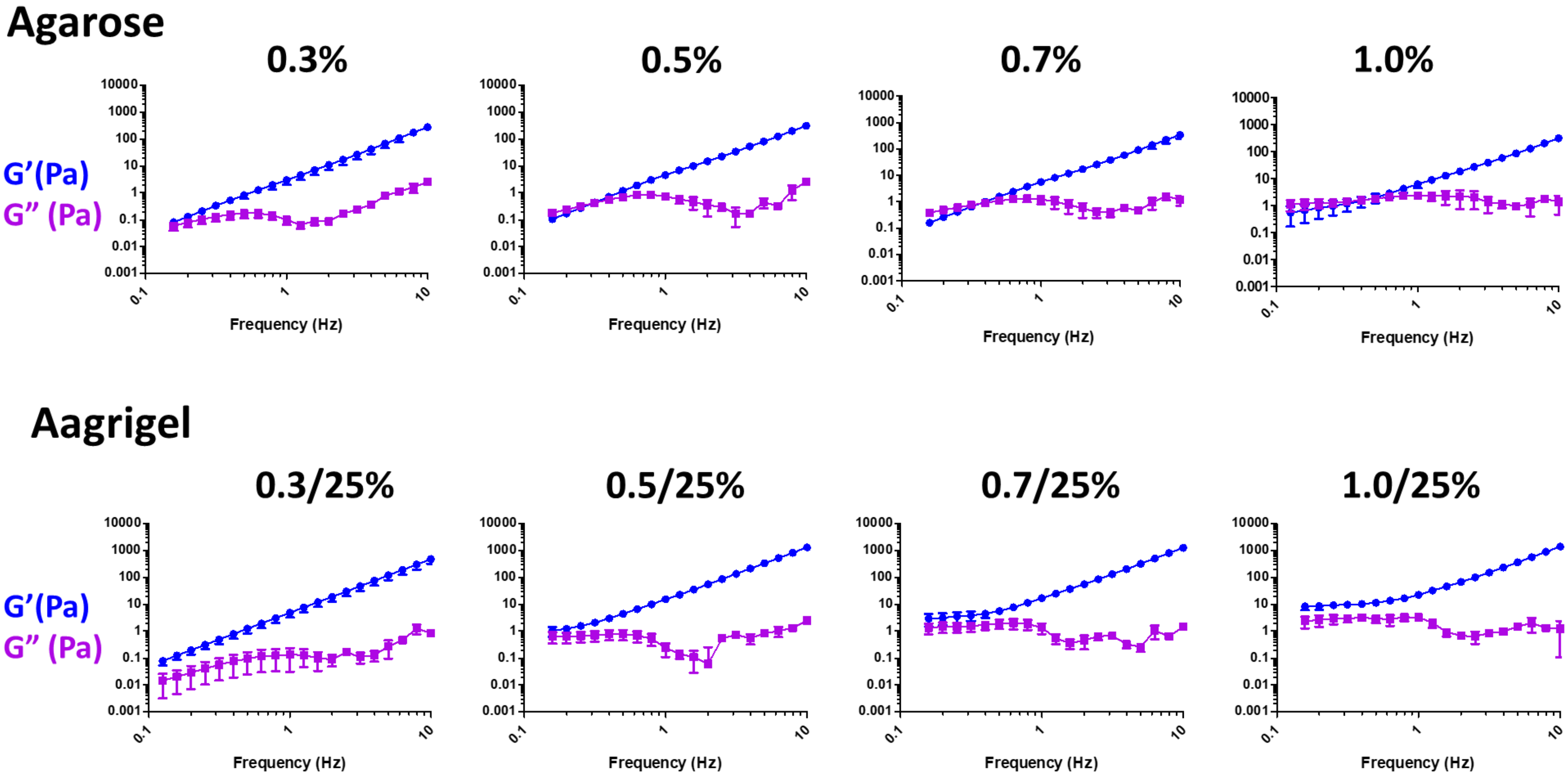

Figure S2. Viscoelastic Properties of agarose and agrigel scaffold at different concentrations Related to Figure 5: Storage modulus (G') and loss modulus G" of different agarose or agrigel (agarose/matrigel) mixtures. Results are presented as mean±SEM of n=3 independent experiments.

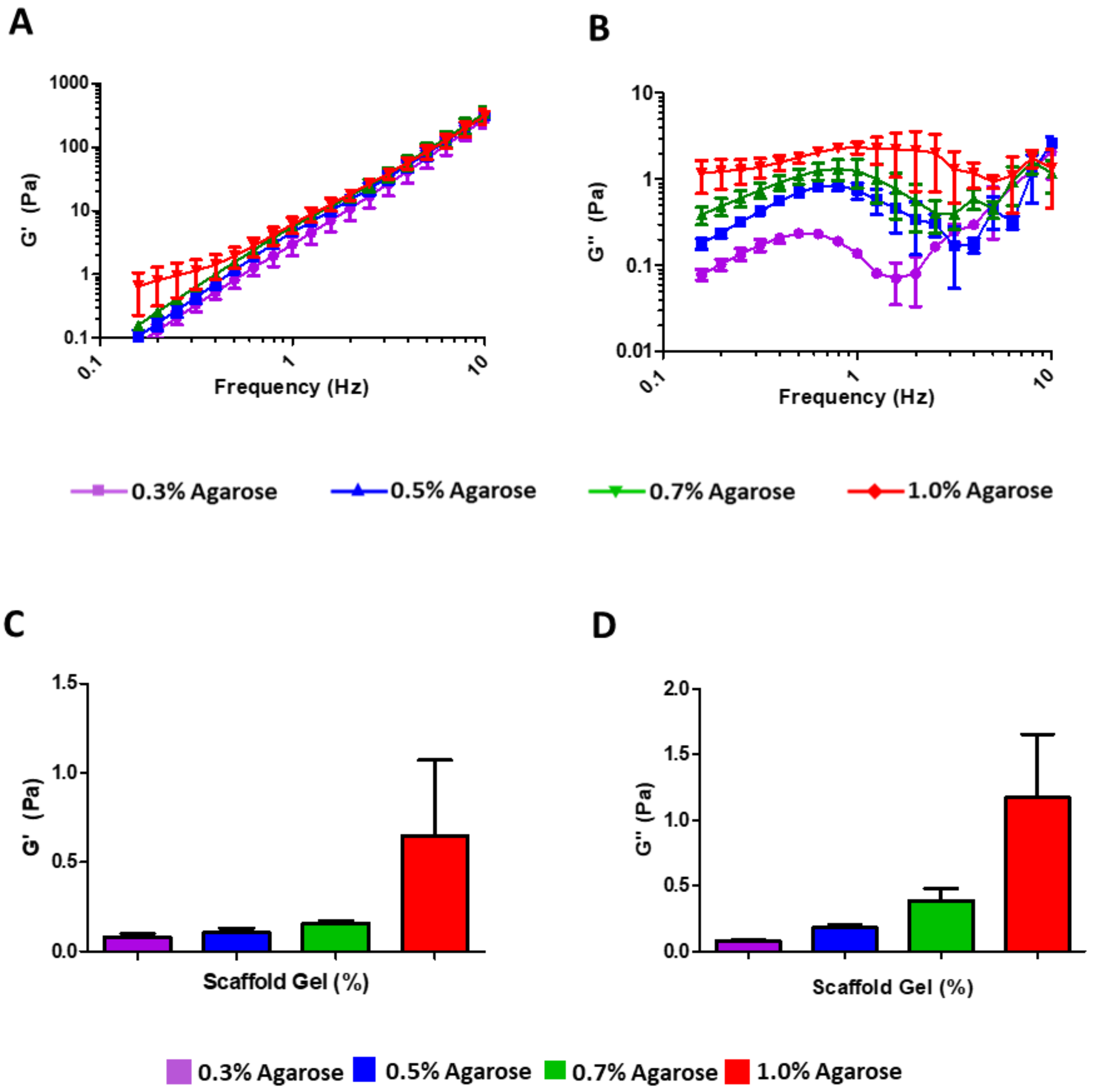

Figure S3. Viscoelastic properties of agarose gel scaffold, Related to Figure 5-7: (A) Storage modulus (G') and loss modulus G" (B) of agarose at different concentrations. (C) Storage modulus (G') and (D). loss modulus (G") of agarose at different concentrations measured at 0.16Hz. Results are presented as mean±SEM of n=3 independent experiments.

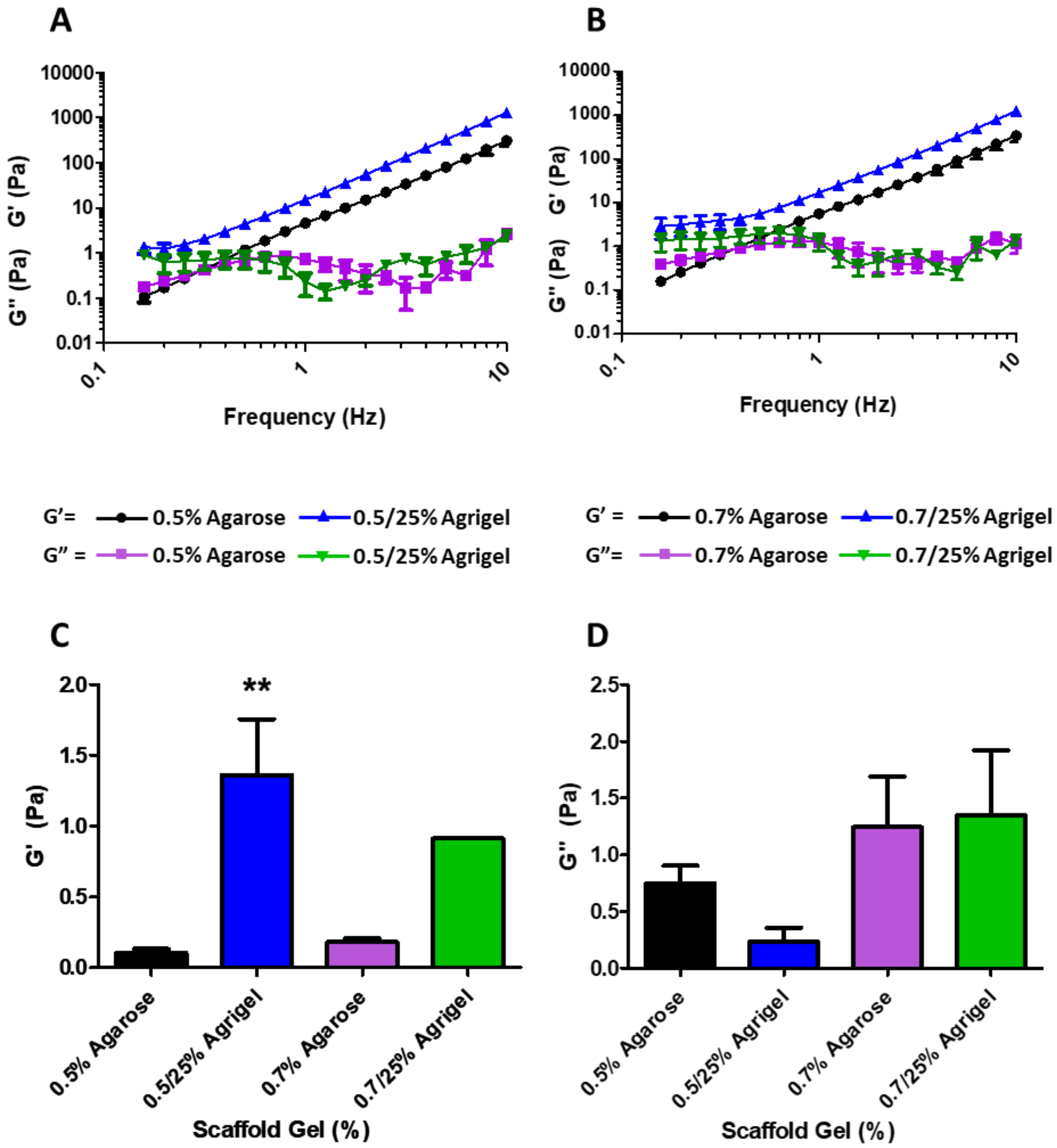

Figure S4. Comparison of viscoelastic characteristics of agarose and agrigel, Related to Figure 5-7: (A) Storage and loss modulus of 0.5% agarose compared to 0.5%/25% agrigel. (B) Storage and loss modulus of 0.7% agarose compared to 0.7%/25% agrigel. (C) Storage modulus of agarose at different concentrations measured at 1Hz. (D) Loss modulus of agarose at different concentrations measured at 1Hz. All data is presented as mean±SEM of n=3 independent experiments using ANOVA with Dunns correction compared to agarose. *p<0.05, **p<0.01, ***p<0.001.

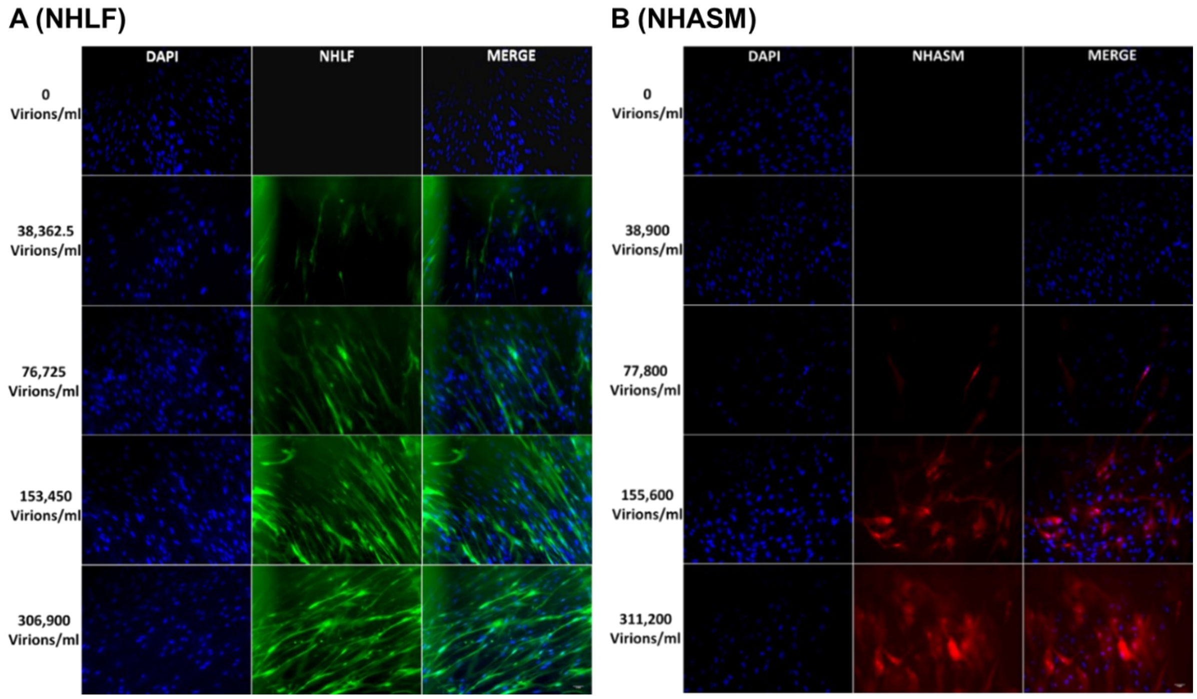

Figure S5. Stromal Cell Lentiviral Transduction, Related to Figure 7 and 8: 100, 000 normal healthy human lung fibroblasts (NHLF) and normal healthy human airway smooth muscle (NHASM) cells were transfected with 306,900 YFP and 311,200 mCherry viral particles respectively in 1ml media with a multiplicity of infection (MOI) = 3. Images are at 5x magnification, Scale bar is 50µm. Images are representative of those from n=3 independent experiments.

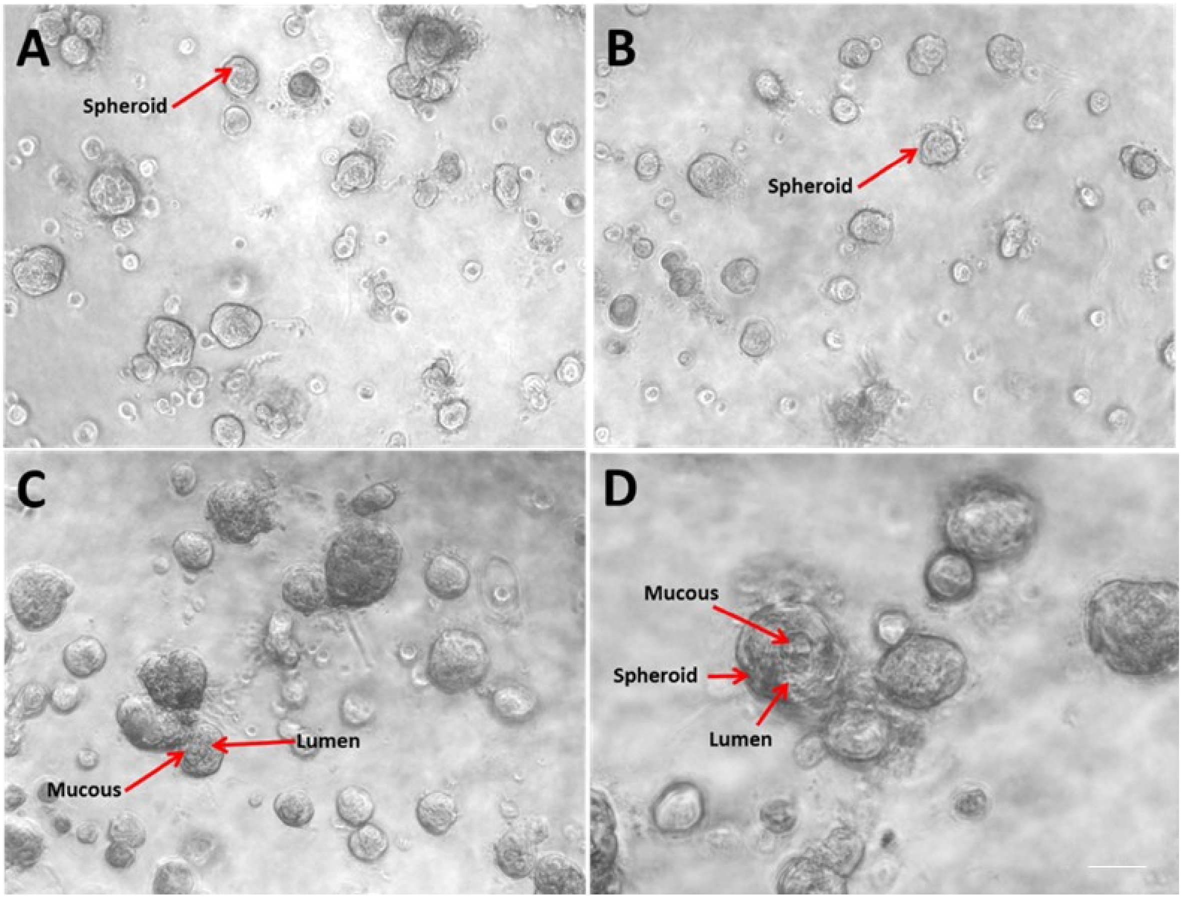

Figure S6. Epithelial cell behaviour on gel surface, Related to Figure 1: Epithelial cells that did not migrate into the gel formed into spheroids and did not form rod-like or tubular structures. Images are representative of those seen in 2 wells per plate and for n=3 donors. Scalebar = 100µm
